# Microcalcification and Thoracic Aortopathy: A Window Into Disease Severity

**DOI:** 10.1161/ATVBAHA.122.317111

**Published:** 2022-06-30

**Authors:** Alexander J. Fletcher, Jennifer Nash, Maaz B.J. Syed, Mark G. Macaskill, Adriana A.S. Tavares, Niki Walker, Hannah Salcudean, Jonathon A. Leipsic, Kelvin H.H. Lim, Jillian Madine, William Wallace, Mark Field, David E. Newby, Rihab Bouchareb, Michael A. Seidman, Riaz Akhtar, Stephanie L. Sellers

**Affiliations:** British Heart Foundation Centre for Cardiovascular Science (A.J.F., J.N., M.B.J.S., N.W., D.E.N.), University of Edinburgh, United Kingdom.; Edinburgh Imaging Facility, Queens Medical Research Institute (M.G.M., A.A.S.T.), University of Edinburgh, United Kingdom.; Division of Pathology (W.W.), University of Edinburgh, United Kingdom.; Department of Child Health, University of Glasgow, School of Medicine and Dentistry, United Kingdom (A.J.F.).; Scottish Adult Congenital Cardiology Service, Golden Jubilee National Hospital, Clydebank, Glasgow, United Kingdom (N.W.).; Department of Radiology, Division of Cardiology, Cardiovascular Translational Lab at the Centre for Heart Lung Innovation, St. Paul’s Hospital and University of British Columbia, Vancouver, Canada (H.S., J.A.L., S.L.S.).; Department of Cardiothoracic Surgery, Royal Infirmary of Edinburgh, United Kingdom (K.H.H.L.).; Institute of Systems, Molecular and Integrative Biology, Faculty of Health and Life Sciences (J.M., M.F., R.A.), University of Liverpool, United Kingdom.; Liverpool Centre for Cardiovascular Sciences (J.M.), University of Liverpool, United Kingdom.; Department of Cardiothoracic Surgery, Liverpool Heart and Chest Hospital (LCCS), United Kingdom (M.F.).; Department of Medicine, Cardiovascular Research Institute, Icahn School of Medicine at Mount Sinai, New York, NY (R.B.).; Department of Laboratory Medicine and Pathobiology, Toronto General Hospital, Canada (M.A.S.).; Department of Mechanical, Materials and Aerospace Engineering, School of Engineering, University of Liverpool, United Kingdom (R.A.).

**Keywords:** aneurysm, dissecting, aortic aneurysm, aorta, thoracic, calcinosis, elastin, sodium fluoride, vascular calcification

## Abstract

Patients with thoracic aortopathy are at increased risk of catastrophic aortic dissection, carrying with it substantial mortality and morbidity. Although granular medial calcinosis (medial microcalcification) has been associated with thoracic aortopathy, its relationship to disease severity has yet to be established.

**Methods:**

One hundred one thoracic aortic specimens were collected from 57 patients with thoracic aortopathy and 18 control subjects. Standardized histopathologic scores, immunohistochemistry, and nanoindentation (tissue elastic modulus) were compared with the extent of microcalcification on von Kossa histology and 18F-sodium fluoride autoradiography.

**Results:**

Microcalcification content was higher in thoracic aortopathy samples with mild (n=28; 6.17 [2.71–10.39]; *P*≤0.00010) or moderate histopathologic degeneration (n=30; 3.74 [0.87–11.80]; *P*<0.042) compared with control samples (n=18; 0.79 [0.36–1.90]). Alkaline phosphatase (n=26; *P*=0.0019) and OPN (osteopontin; n=26; *P*=0.0045) staining were increased in tissue with early aortopathy. Increasingly severe histopathologic degeneration was related to reduced microcalcification (n=82; Spearman ρ, −0.51; *P*<0.0001)—a process closely linked with elastin loss (n=82; Spearman ρ, −0.43; *P*<0.0001) and lower tissue elastic modulus (n=28; Spearman ρ, 0.43; *P*=0.026).^18^F-sodium fluoride autoradiography demonstrated good correlation with histologically quantified microcalcification (n=66; r=0.76; *P*<0.001) and identified areas of focal weakness in vivo.

**Conclusions:**

Medial microcalcification is a marker of aortopathy, although progression to severe aortopathy is associated with loss of both elastin fibers and microcalcification.^18^F-sodium fluoride positron emission tomography quantifies medial microcalcification and is a feasible noninvasive imaging modality for identifying aortic wall disruption with major translational promise.

**Highlights**

- Increased medial microcalcification is found in patients with mild and moderate histopathologic thoracic aortic aneurysm disease and is associated with higher levels of alkaline phosphatase and osteopontin.
- Severe histopathologic thoracic aortopathy is associated with low levels of microcalcification, related to total loss of elastin fibers on which the microcalcification has precipitated, as well as reduced tissue elastic modulus.
- Medial microcalcification can be detected and quantified accurately using the radiotracer ^18^F-sodium fluoride, which has major promise in the noninvasive detection of thoracic aortic aneurysm wall severity.

Thoracic aortic aneurysm is a sinister condition, remaining clinically silent until the point of catastrophic dissection or rupture, with only 32% of patients surviving such an event.^1^ The risk of these catastrophic complications increases with maximum aortic aneurysm diameter,^2^ and current management strategies advocate replacement of the aorta once the risk of aortic complications outweighs the risks of elective surgery. International guidelines outline threshold diameters for elective repair, which are adjusted for certain risk factors, such as hypertension, bicuspid aortic valve, and connective tissue disease.^3,4^ Despite this, over 70% of patients who experience aortic dissection do so below current diameter thresholds.^5,6^ Novel non–size-dependent strategies are, therefore, urgently needed to identify patients with thoracic aortic aneurysms that are vulnerable to these potentially fatal complications.

**See cover image**

Aortic wall microcalcification, or granular medial calcinosis, is an underappreciated disease process that represents the deposition of hydroxyapatite crystals formed of calcium phosphate within the extracellular matrix. Microcalcification has been described in thoracic aneurysms of various etiologies^7,8^ and is specifically associated with elastin fragmentation, vascular smooth muscle cell phenotypic switching, and increased aortic wall rupture risk on biomechanical testing.^8–10^ We have previously shown that abdominal aortic aneurysm microcalcification can be detected using^18^F-sodium fluoride positron emission tomography, and its uptake correlates with aneurysm expansion rate, as well as the risk of subsequent complications.^11^ A better understanding of the relationship between thoracic aortic aneurysm microcalcification,^18^F-sodium fluoride positron emission tomography, pathological mechanisms, and disease severity has the potential to uncover novel therapeutic targets and act as a clinical tool to diagnose and to monitor thoracic aortopathy.

Using histology and^18^F-sodium fluoride autoradiography, we here aimed to assess the relationship between aortic wall microcalcification and thoracic aortopathy and aneurysm disease. We sought to investigate the pathways associated with aneurysm microcalcification and assess the relationship between microcalcification, tissue biomechanical properties, and histological disease severity.

## Methods

### Human Ascending Aortic Tissue

**Figure 1.**
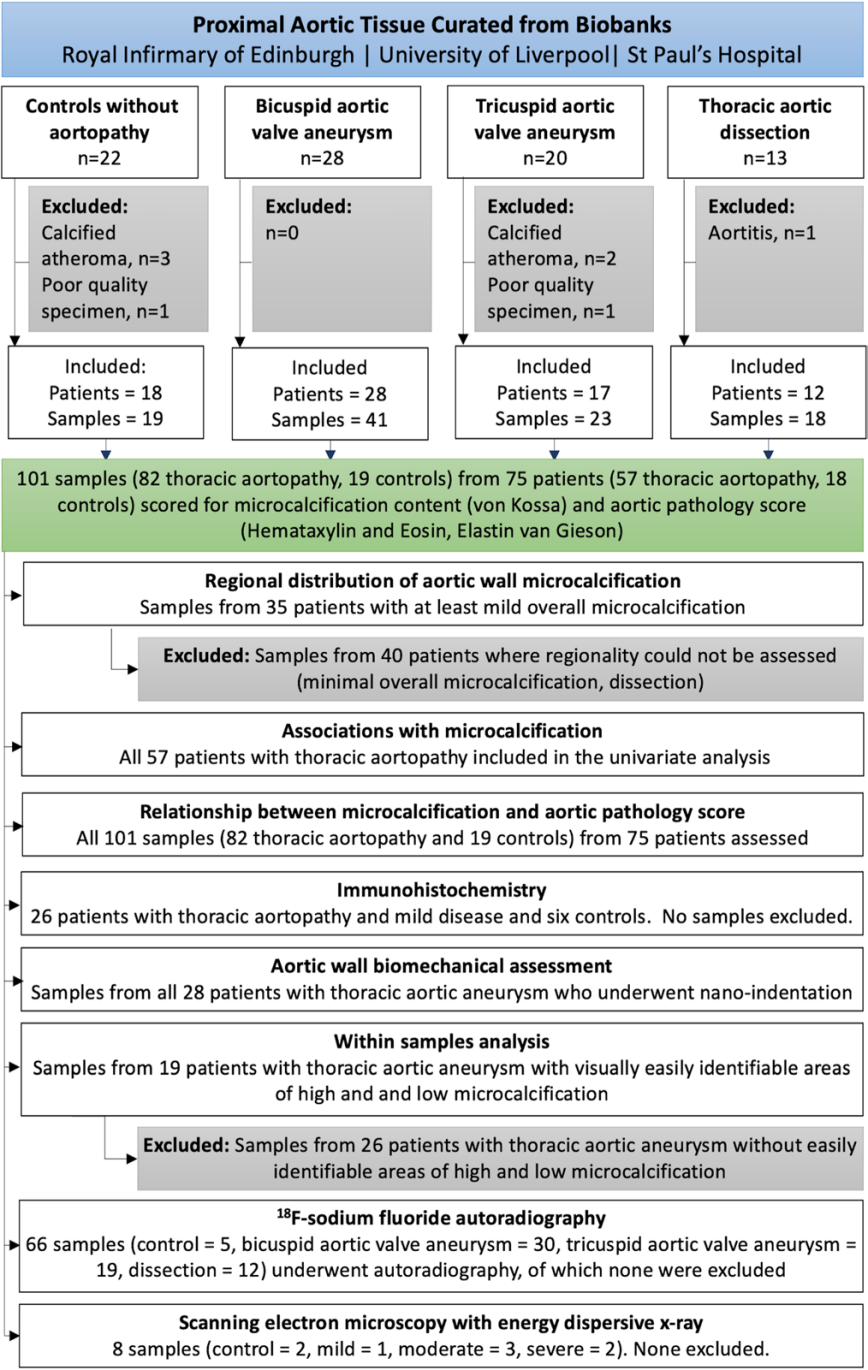
**Study flowchart.** A summary of the sample collected, individual experiments, and samples excluded.

Aneurysmal thoracic aortic tissue was obtained from patients with bicuspid (n=28) or tricuspid (n=20) aortic valves undergoing elective repair of aneurysm meeting the American Heart Association or European Society of Cardiology guidelines.^3,12^ Aortic tissue was also retained from the flap and true lumen of patients undergoing surgery for acute or chronic aortic dissection (n=13). Control aortic tissue samples were obtained from patients undergoing proximal vein graft anastomosis during coronary artery bypass grafting (n=9) or donor heart transplant aortic trimmings (n=13). Specimens were obtained from biobanks at 3 separate institutions: the Royal Infirmary of Edinburgh, United Kingdom; the Cardiovascular Tissue Registry at the University of British Columbia and St. Paul’s Hospital Centre for Heart Lung Innovation, Vancouver, Canada; the University of Liverpool, United Kingdom (for overview of methodology, see Figure 1). Samples at each institution were collected from consenting patients or relatives and in accordance with ethical approvals (15/ES/0094, 18/SS/0136, 14/NW/1212, 19-09 Liverpool Bio-Innovation Hub project approval and H21-00987–Providence Health Care/UBC Research Ethics Board). Study data that support the findings of this study are available from the corresponding author upon reasonable request.

### Tissue Sample Preparation

Aortic aneurysm specimens were obtained at the time of elective surgical repair. Aortic tissue from patients with a dissection was taken from the entry tear or nearby true lumen. A subset of aortic aneurysm samples was snap-frozen to enable accurate biomechanical analysis, after which they were paraffin embedded in the axial plane. All other samples were immediately fixed in 4% paraformaldehyde for at least 24 hours before a 5-mm sample of aneurysmal aortic tissue was cut and paraffin embedded in the axial plane. Transplant donor samples were fixed in 10% neutral buffered formalin immediately following collection at time of explantation and subsequently embedded in paraffin. Sections were embedded in the axial plane to generate an aortic wall cross section.

### Histological Assessment

Histology was performed by 2 institutions (NHS Lothian Clinical Pathology Laboratory and University British Columbia) according to local protocols. One to 4 paraffin-embedded sections were sliced at 5 to 6 µm per sample and slide mounted. Samples containing macrocalcified atheroma requiring decalcification or poor-quality specimens (freezing or imaging artifact) precluding histological grading were excluded. Histological staining included von Kossa, hematoxylin and eosin, elastin van Gieson, and Movat pentachrome performed according to local protocols. All images were captured using the Axioscan slide scanner (Zeiss, Germany) and analyzed using the FIJI software (v 2.0.0, open source) or Aperio Slide Scanner using the ImageScope software (Leica Biosystems, Germany). Histopathologic severity scoring was performed by an experienced pathologist (W.W.) blinded to the results of microcalcification quantification and in accordance with international consensus guidelines.^13^ Briefly, aortic histology was split into 5 major categories; intralamellar mucoid extracellular matrix accumulation, translamellar mucoid extracellular matrix accumulation, elastin fiber fragmentation or loss, smooth muscle cell nuclei loss, and laminar medial collapse. Each category is split into absent, mild, moderate, or severe, scoring 0, 1, 2, or 3 points, respectively. The final score represents a sum of each category with possible scores ranging from 0 to 15. The presence or absence of atheroma was also recorded.

### Scanning Electron Microscopy and Dispersive Radiograph Energy

Paraffin sections were deparaffinized using Xylene followed by ethanol 100%. Slides were then completely dried out before coating them with carbon. Scanning electron microscopy with secondary electron was used to visualize the tissue. Dispersive radiograph energy coupled with the electron beam of the scanning electron microscopy was used as a chemical analysis method to determine the elemental composition of the mineral deposition on the tissue.

### Immunohistochemistry

Immunohistochemistry was performed on 26 samples from 13 patients with aortopathy and 6 control subjects. Scoring was completed from high-resolution slide scans. Immunohistochemistry was completed using OPN (osteopontin; catalog No. 07264; 1:100 dilution; Sigma-Aldrich), alkaline phosphate (AP; ab95462 1:300; Abcam), BMP2 (bone morphogenic protein 2; AHP960 1:100; Biorad), WNT3A (wingless-type MMTV integration site family member 3a; ab28472; Abcam), cleaved caspase 3 (No. 9664; 1:100 dilution; Cell Signaling), and p-ERK (phospho-extracellular signal-related kinase; 9102S, 1:50 dilution; Cell Signaling). Staining was performed via automated staining with a Leica Bond Rx system using Bond Epitope Retrieval Solution 1 (pH=6, catalog No. AR9961) and Bond Polymer Refine Red Detection (catalog No. DS9390). Background staining was assessed via assessment of sections processed with the omission of primary antibody and the consideration of normal human aortic background from normal transplant donors. The omission of the primary antibody served as negative controls. Blinded qualitative categorization of staining was performed by a pathologist (S.S.) using a 4-point classification based on relevant cell positivity as described previously in target tissue^14^: 0, no notable staining; 1, 5% to 20% of relevant cells are weakly positive; 2, 21% to 50% of relevant cells are positive; 3, 51% to 100% (see Figure S1 for representative staining of OPN, AP, and cleaved caspase III as examples).

### Immunofluorescence

To assess the RUNX2 (Runt-related transcription factor 2) expression, we used immunofluorescence on paraffin sections. First, sections were treated with xylene twice 10 minutes and then rehydrated twice in 10 minutes using serial baths of 100% followed by 5-minute baths in 95%, 70%, and 50% each. Slides were then rinsed with water for 10 minutes. Citrate was used to retrieve antigen for 30 minutes. Sections were blocked using goat serum for 1 hour. Primary antibody was diluted 50-fold in dilution buffer; slides were then incubated overnight at 4 °C. Slides were washed 3× in PBS 1× and then incubated with Alexa red secondary antibody for 1 hour. Sections were then washed with PBS 1× 5 minutes each. After that, sections were mounted with DAPI (4’, 6-diamidino-2-phenylindole) and imaged using epifluorescence microscope. Image Quantifications: NIH Image J software was used to quantify the mean fluorescence of RUNX2.

### Histological Quantification

As multiple samples from the same patient were scored in some instances, the sample with the highest pathology score was used for patient-level analysis, in line with international consensus.^13^ Microcalcification quantification was performed by a trained user (A.J.F.) blinded to the histological scoring, immunohistochemistry, and nanoindentation. von Kossa images were transformed to 8-bit gray scale, a threshold value set to 50% of total opacity of the media, and altered by no more than 10% to produce optimal visual coverage of microcalcification. A region of interest was drawn around the intima and media, and the microcalcification concentration reported as the percentage area over threshold values. The percentage area of microcalcification was subdivided into minimal (0%–0.99%), mild (1%–4.99%), moderate (5%–9.99%), or severe (≥10%) categories (for examples, see Figure S2). The intraobserver variability of microcalcification percentage area calculations was determined in 24 randomly selected samples. Analyses were performed twice in random order by a single trained user (A.J.F.) at least 2 months apart to minimize recall bias and blind to the original analysis. To assess the distribution of microcalcification, regions of interest were drawn around the intima, inner media (luminal 50%), and outer media (adventitial 50%). To allow comparison across samples with varying overall microcalcification content, a ratio between each area and the total microcalcification uptake of each sample was reported.

Elastin concentration was determined using a similar method. The elastin van Gieson image was transformed to gray scale 8-bit, with the threshold set using the Otsu formula and altered visually within 20% of the Otsu value to achieve optimal elastin opacity. Wall thickness was measured from the intima to the external elastic lamina. Smooth muscle cell density was measured on the hematoxylin and eosin–stained tissue, which was transformed to 8-bit gray scale. The threshold was set using the Otsu formula and altered within 20% to achieve the visually optimal signal-to-noise ratio for cell nuclei. Using the inbuilt cell counter function, the cell density was calculated as the number of cells in a region of interest divided by the area. In a subset of samples with clear areas of high and low microcalcification, mean wall thickness, elastin concentration, and nuclei cell density were calculated using 3 full thickness regions of interest in both high and low microcalcification concentration areas. Samples with absent microcalcification content, or homogenous distribution (no distinct areas of high or low microcalcification), were excluded from this subanalysis.

### Nanoindentation

Oscillatory nanoindentation allows the assessment the elastic and viscous properties of localized regions of the tissue by measuring the shear storage (G′) and shear loss modulus (G″), respectively. G′ is directly related to the tissue elastic modulus (E).^15^ Small indentations of 0.5 µm at a frequency of 110 Hz were performed using a 100-μm-flat punch indenter tip (Synton-MDP, Ltd, Nidau, Switzerland) with a G200 nanoinderter equipped with a DCM-II actuator (KLA-Tencor, Milpitas) by an experienced investigator (R.A.) as described previously.^16^

### Biochemical Assessment

Elastin, collagen, and glycosaminoglycan content were assessed using established protocols as described previously.^16^ Briefly, aortic tissue was digested with papain (for collagen and glycosaminoglycan analysis) or oxalic acid (for elastin analysis). Following digestion, collagen content was determined by measuring hydroxyproline concentration in the tissue using the 1,3-dimethylbutylamine dye, glycosaminoglycan content was measured using the dimethyl methylene blue assay, and elastin was measured using Fastin Elastin Kit (Biocolor, County Antrim, United Kingdom).

### Ex Vivo ^18^F-Sodium Fluoride Autoradiography

Formalin-fixed paraffin-processed sections were rehydrated and equilibrated in PBS for 30 minutes. Sections were then incubated with 100 kBq/mL of ^18^F-sodium fluoride in PBS for 1 hour at room temperature with a blocking control (10 µmol/L sodium fluoride) before two 5-minute washes in PBS and one in deionized water. Dried sections were exposed to a high-resolution autoradiography plate (BAS-IP-SR 2040; Cytiva), which were imaged on an autoradiography imager (Amersham Typhoon IP Biomolecular Imager, Cytiva).

Sample ^18^F-sodium fluoride content was quantified using the FIJI software (v 2.0.0, open source). Gray scale images were imported and a region of interest drawn around the perimeter of the image to provide mean background activity. Regions of interest were drawn around individual samples providing a mean gray intensity. The result was adjusted by dividing by the background activity, standardizing measurements, and allowing samples across separate experiments to be compared.

### In Vivo ^18^F-Sodium Fluoride Positron Emission Tomography Example

We include an example ^18^F-sodium fluoride positron emission tomography scan (Biograph mCT; Siemens Healthcare, Germany) fused with a magnetic resonance imaging angiogram (Biograph mMR; Siemens Healthcare) taken from an ongoing clinical trial; the assessment of risk in thoracic aortic disease using ^18^F-sodium fluoride study (https://www.clinicaltrials.gov; unique identifier: NCT04083118).

### Statistical Analysis

All statistical analyses were performed in RStudio (V1.3.959, general common license). Categorical variables were presented as number (percentage). Continuous variables with normal distribution are presented as mean±SD, whereas non-normally distributed variables were presented as median (interquartile interval). Analyses of variable influence on patient-level specimen microcalcification content were performed using a univariable linear regression. When comparing between groups, both overall (Kruskal-Wallis) and individual (Wilcox) nonparametric tests were used. For correlations between numeric variables, Spearman rank-sum test was used. For within-sample analysis, paired Wilcox tests were used. A 2-sided *P*<0.05 was considered statistically significant.

## Results

### Study Population

Specimens from 8 participants were excluded from analysis (5 macrocalcified atheromas, 2 freezing or imaging artifacts, and 1 aortitis), leaving specimens from 75 participants (57 patients with thoracic aortopathy and 18 control subjects) for assessment. Participants with bicuspid aortic valve aneurysms tended to be younger although the prevalence of valvular heart disease, cardiovascular risk factors, and maximal aortic diameters were similar between the 3 aortopathy subgroups (Table). Those with dissection had more severe histopathologic degeneration scores than those with bicuspid or tricuspid valve thoracic aortic aneurysms (χ^2^ *P*=0.001; Table S1).

**Table.**
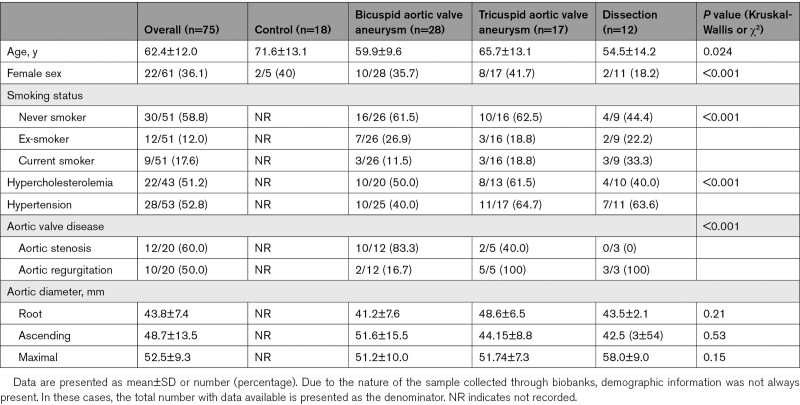
Patient Demographics Subdivided Into Disease Subgroups and Control Subjects

### Aortic Microcalcification

The microcalcification quantification method demonstrated excellent intraobserver repeatability, with a mean absolute percentage area difference of 0.44%, a coefficient of repeatability of 4.5%, and a interclass correlation coefficient of 0.97 (0.93–0.99; Figure S3). When considered as a categorical variable, the repeatability was similarly excellent with 20 of 24 (83%) given the same grade (interclass correlation coefficient, 0.9 [0.78–0.95]; Table S2).

**Figure 2.**
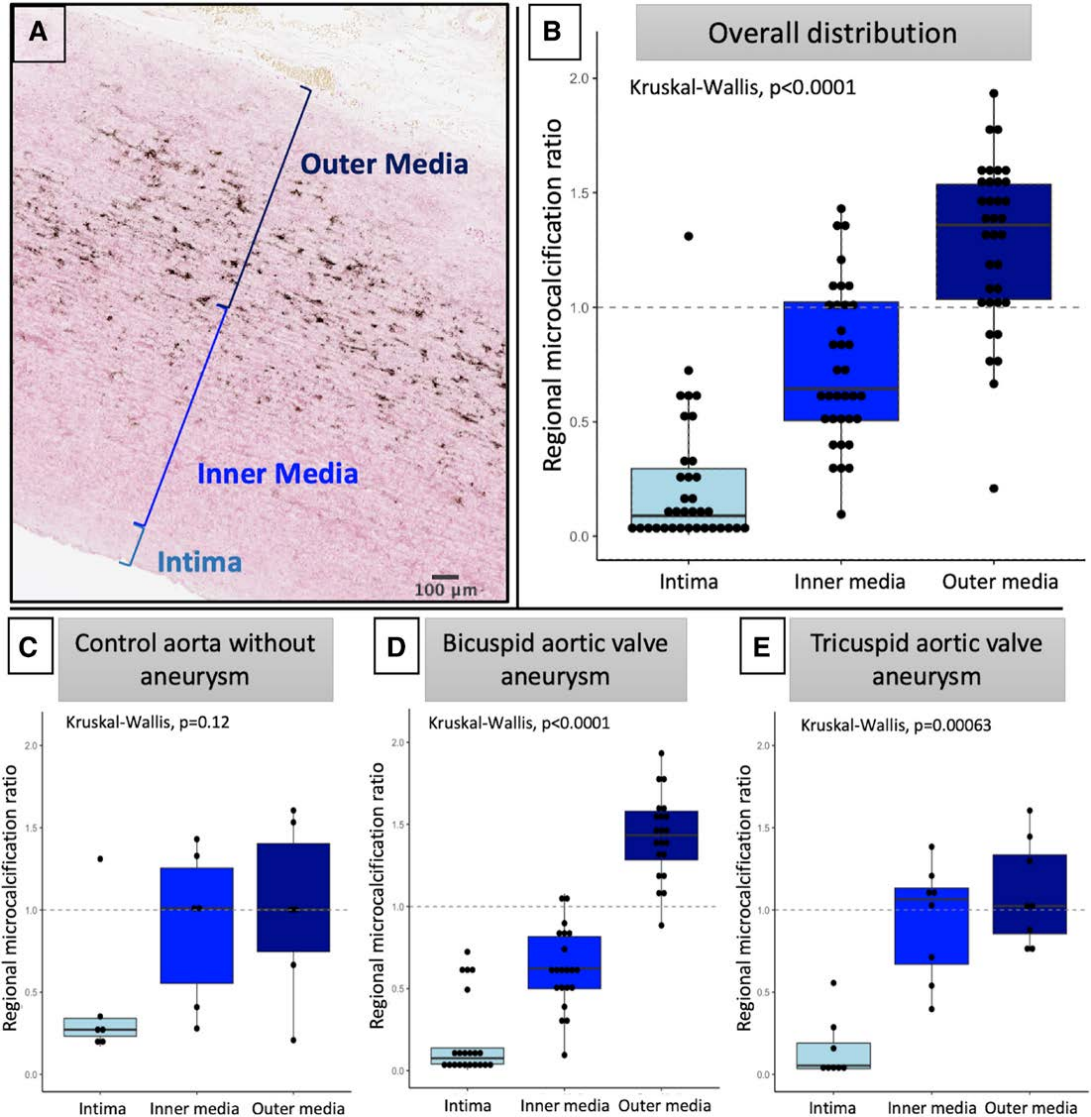
**Distribution of microcalcification in the proximal aortic wall of aneurysms.** Assessment of the pattern of microcalcification across the aortic wall. **A**, Representative image of aortic wall stained with von Kossa, demonstrating significant microcalcification in the media, particularly the outer media, but not the intima. **B**, Across all samples assessed, as a ratio of total sample uptake, there was relatively little microcalcification of the intima, whereas there was significant deposition in the media, particularly the outer media. **C** through **E**, This pattern was consistent across aneurysm and control samples, with bicuspid aortic valve demonstrating a particularly strong preponderance for microcalcification in the outer media.

Overall, 43 (57%) of the 75 specimens had at least mild medial microcalcification on von Kossa staining, with only 1 (1.3%) specimen having intimal microcalcification associated with complex atheroma. Microcalcification was clearly localized to the media, with particularly intense microcalcification seen in the outer media (outer media/total sample microcalcification content ratio, 1.36 [1.03–1.54]), but there was very little staining in the intima (intima/total sample microcalcification content ratio, 0.09 [0.04–0.30]; Figure 2).

In 8 specimens with varying degrees of aortic histopathologic disease severity (2 control, 1 mild, 3 moderate, and 2 severe), scanning electron microscopy with dispersive radiograph energy confirmed that the microcalcification was crystalized calcium phosphate. Control samples appeared to have little microcalcification, while those with mild or moderate aortopathy had deposition of microcalcification along intact elastin (Figures S4 and S5). Those with severe histopathologic aortopathy had large areas of mucoid extracellular matrix accumulation devoid of elastin or microcalcification (Figure S5).

**Figure 3.**
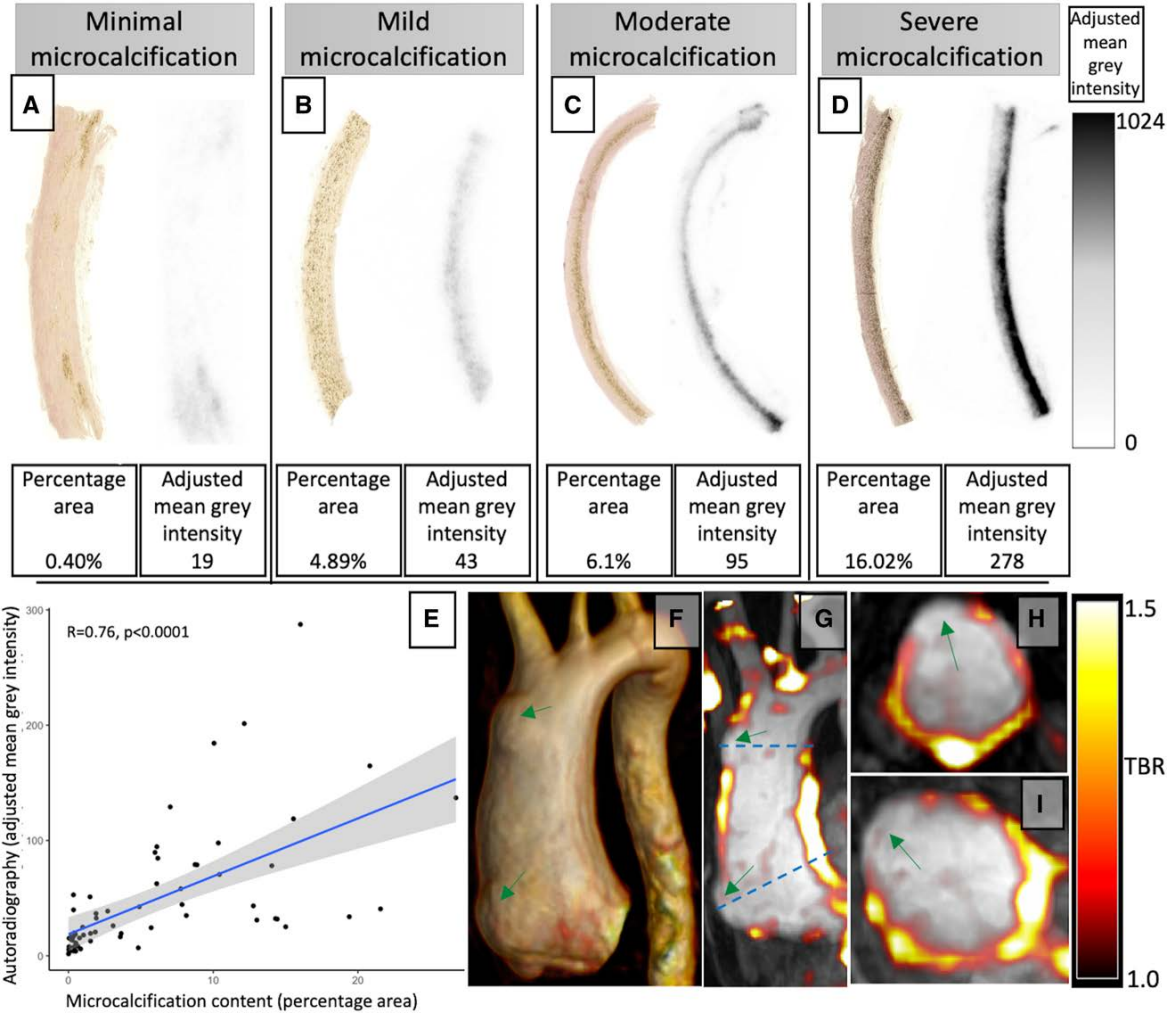
**18F-sodium fluoride is an excellent marker of medial microcalcification. A** through **D**, Visual comparison of samples with minimal (**A**), mild (**B**), moderate (**C**), and severe (**D**; scale threshold applies to all autoradiography images). There was a good correlation between histologically assessed von Kossa, and ^18^F-sodium fluoride determined microcalcification in a sample-level analysis (**E**). In vivo ^18^F-sodium fluoride positron emission tomography fused with magnetic resonance angiogram in a patient with bicuspid aortic valve and saccular aneurysm of the ascending aorta seen on 3D reconstruction (**F**). Fused angiogram and ^18^F-sodium fluoride (**G–I**). Note reduced ^18^F-sodium fluoride at sites of aortic wall expansion (green arrows). TBR indicates tissue to background ratio.

Autoradiography with ^18^F-sodium fluoride colocalized precisely with microcalcification on von Kossa staining, demonstrating a good correlation (Spearman ρ, 0.76; *P*<0.0001; Figure 3). Furthermore, to demonstrate the feasibility of using ^18^F-sodium fluoride to assess aortopathy in vivo, we present the case of a 40-year-old with bicuspid aortic valve and aortopathy with focal aortic wall outpouchings who underwent combined ^18^F-sodium fluoride positron emission tomography and magnetic resonance imaging angiography (Figure 3). There was high overall uptake in the ascending aorta, with reduced uptake in the areas of focal expansion.

### Rise and Fall of Medial Microcalcification With Progressive Aortopathy

**Figure 4.**
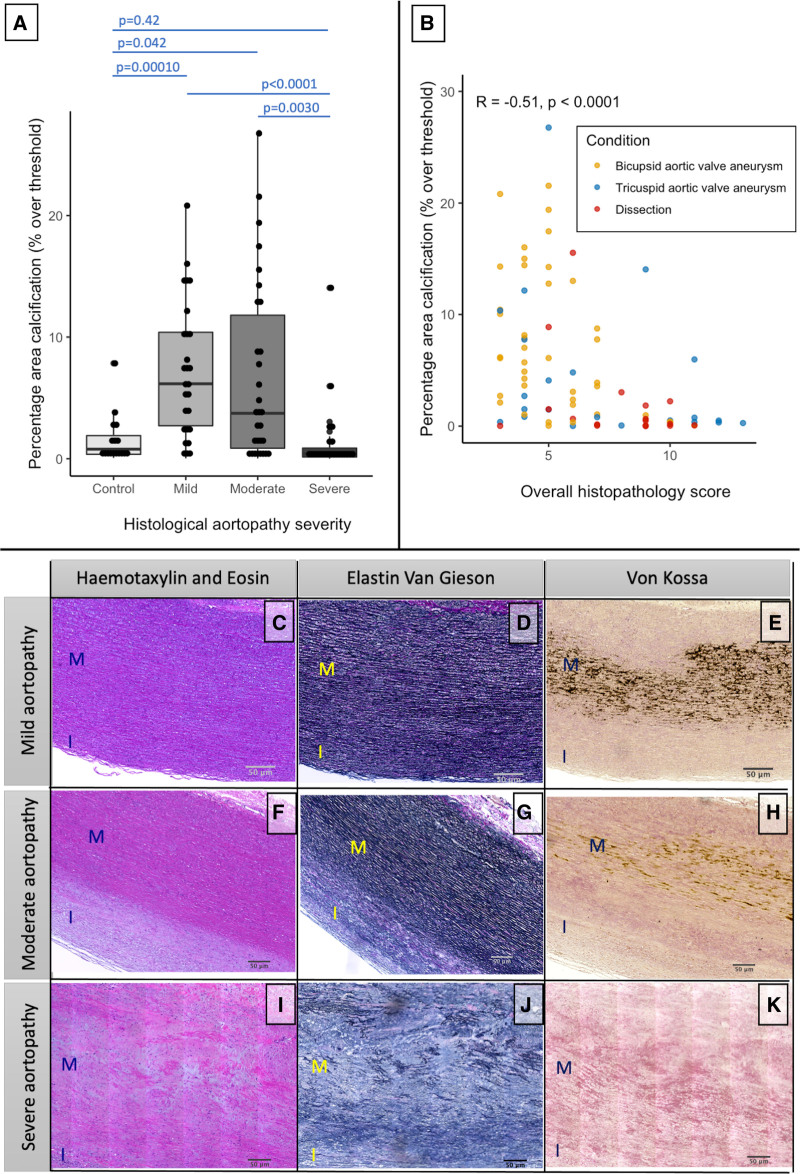
**The relationship between aortopathy severity and microcalcification. A**, Patients with mild (Wilcox with Bonferroni correction, *P*=0.00010) or moderate (Wilcox with Bonferroni correction, *P*=0.042) histopathologic aortopathy severity had more microcalcification than control samples. Those with severe disease had less microcalcification content than either mild (Wilcox with Bonferroni correction, *P*<0.0001) or moderate (Wilcox with Bonferroni correction, *P*=0.0030). **B**, In aortopathy samples, there was an inverse relationship between histopathologic disease severity and microcalcification content (Spearman ρ, −0.51; *P*<0.0001). **C** through **K**, Representative examples of hematoxylin and eosin (**C**, **F**, and **I**), elastin van Gieson (**D**, **G**, and **J**), and von Kossa (**E**, **H**, and **K**) in mild, moderate, and severe histopathologic disease, with minimal microcalcification coinciding with near total elastin loss in severe disease.

In 82 thoracic aortopathy specimens, aortic pathology score was categorized into mild (pathology score, 2–4), moderate (pathology score, 5–7), and severe (pathology score, ≥8) disease (thresholds determined using pathology score tertiles). Compared with control specimens (0.79 [0.36–1.90]), microcalcification content was higher in those with mild (6.17 [2.71–10.39], Wilcox with Bonferroni correction *P*=0.00010) or moderate (3.74 [0.87–11.80], Wilcox with Bonferroni correction *P*<0.042) aortic pathology scores but was similar in those with severe aortic pathology score (0.40 [0.15–0.87], Wilcox with Bonferroni correction, *P*=0.42; Figure 4). In samples from patients with aortopathy, there was an inverse correlation between microcalcification and the overall pathology score (Spearman r, −0.51; *P*<0.0001; Figure 4).

**Figure 5.**
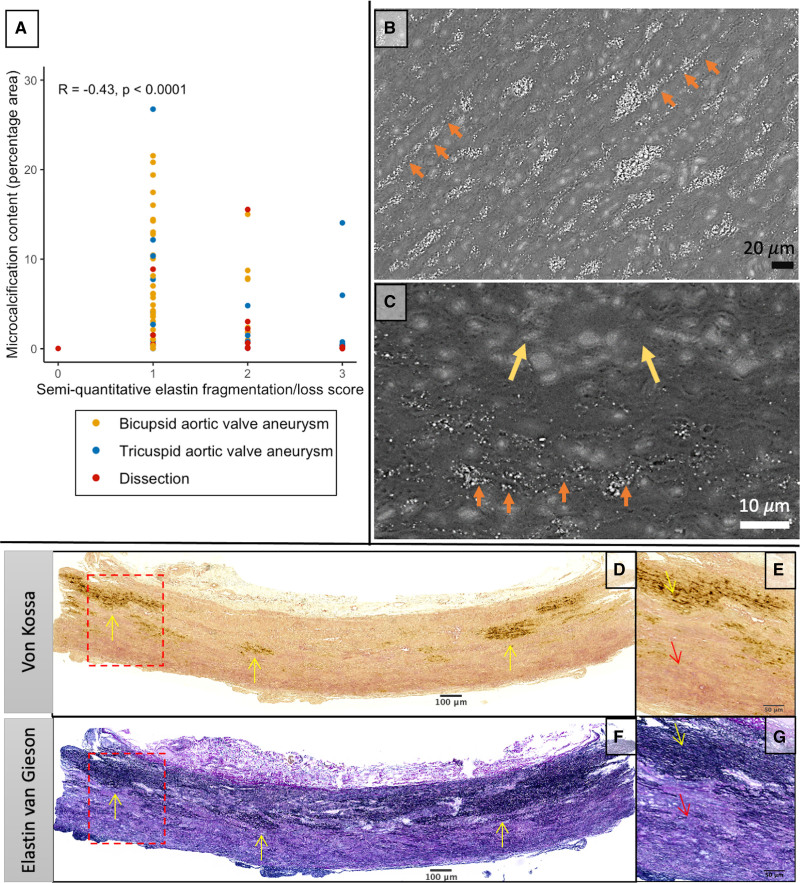
**Elastin fiber loss is associated with reduced microcalcification in severe aortopathy. A**, Association between microcalcification content and elastin fragmentation/loss subcategory (Spearman ρ, −0.43; *P*<0.0001). **B** and **C**, Scanning electron microscopy from a sample with moderate histopathologic severity (**B**) with clear microcalcification precipitation on intact elastin fibers (orange arrows) and severe disease (**C**) demonstrating similar deposition along intact elastin but areas devoid of elastin with no microcalcification. **D** through **G**, Representative von Kossa (**E** and **F**) and elastin van Gieson (**G** and **H**) image from a patient with bicuspid aortic valve, severe elastin fiber loss, and microcalcification content of 0.4% percentage area (minimal). The microcalcification is clearly colocalized with areas of remaining intact elastin fibers (yellow arrows), whereas there is no microcalcification in areas devoid of elastin fibers (red arrows).

To understand the processes driving the relationship with pathology scores, we assessed microcalcification content against individual pathological subcategories. Microcalcification content was inversely associated with the severity of elastin fiber fragmentation and loss (Spearman ρ, −0.43; *P*<0.0001; Figure 5), mucoid extracellular matrix accumulation (Spearman ρ, −0.41; *P*=0.002), and medial collapse (Spearman ρ, −0.23; *P*=0.038) but not cell loss (Spearman ρ, −0.17; *P*=0.14). In those with severe disease, any remaining microcalcification colocalized exclusively with areas of residual elastin (Figure 5). In samples with areas of both high and low microcalcification (n=19; Figure S6), elastin content was increased in the areas of high microcalcification (paired Wilcoxon *P*=0.023), with a trend toward increased wall thickness (paired Wilcoxon *P*=0.08), but there was no difference in nuclei density (paired Wilcoxon *P*=0.32). Similar to the histological results, biochemical analysis appeared to demonstrate an association between histological microcalcification content and elastin concentration (Spearman ρ=0.38; *P*=0.052) although this just failed to reach statistical significance. There were no associations with collagen (Spearman ρ, *P*=0.81) or glycosaminoglycan accumulation (Spearman ρ, *P*=0.91).

### Biology of Microcalcification in Aortopathy

To investigate the mechanisms associated with microcalcification precipitation in early disease, expression of markers for osteogenic smooth muscle cell phenotyping (BMP2, WNT3a, OPN, and RUNX2), pyrophosphate degradation (AP), apoptosis (caspase III), and noncanonical transforming growth factor-β pathways (p-ERK) were assessed, comparing control (n=6) with aortopathy samples in the early phase of disease (≤mild aortic pathological severity score and ≤mild microcalcification, n=21). Both AP (Wilcoxon *P*=0.0019) and OPN (Wilcoxon *P*=0.0045) were increased in early-phase aortopathic disease. Further, the areas of high positivity colocalized with areas of high microcalcification (Figure S7). In contrast, positivity of WNT3a (Wilcoxon *P*=0.51), RUNX2 (Wilcoxon *P*=0.075), cleaved caspase III (Wilcoxon *P*=0.051), BMP2 (Wilcoxon *P*=0.21), and p-ERK (Wilcoxon *P*=0.12) was similar to control specimens.

To assess the relationship between microcalcification and tissue elastic modulus, both nanoindentation and microcalcification content assessments were performed on sections from the same sample. There was a positive correlation between the elastic modulus and both microcalcification (Spearman ρ, 0.43; *P*=0.026) and elastin (Spearman ρ, 0.52; *P*=0.0041) content but not collagen (*P*=0.43) or glycosaminoglycan (*P*=0.32) content.

## Discussion

Medial microcalcification is an overlooked pathological process related to thoracic aortic aneurysm disease, with the underlying mechanisms and its relationship to disease severity poorly understood. In the current study, we demonstrate a striking association between mild and moderate thoracic aortic aneurysm disease and microcalcification, which is linked to AP and OPN and an increase in tissue elastic modulus. Intriguingly, thoracic aortopathy samples with severe histopathologic degeneration consistently had low levels of microcalcification, a process associated with elastin fiber loss, suggesting a nonlinear pathobiological course of microcalcification in thoracic aortopathy. Finally, we found that uptake of the radiotracer ^18^F-sodium fluoride colocalized exquisitely with histological microcalcification, thereby providing support for its use as a noninvasive imaging biomarker of aortopathy severity in similar populations.

We found that patients with mild or moderate thoracic aortic aneurysm disease have over 5× more medial microcalcification content than control subjects, which is in line with previously published reports.^7,17^ The processes mediating the initial precipitation of microcalcification on elastin in thoracic aortic aneurysms are incompletely understood and largely noninflammatory, unlike atherosclerotic aortic aneurysm formation or aortitis.^18^ Our experiments confirm hydroxyapatite deposition along intact elastin, rather than at fragmentation points, although molecular damage at these sites cannot be excluded. Further, in both within-sample analysis and biochemical analysis, we find that higher elastin content is associated with higher microcalcification content, consistent with reports from a Matrix Gla protein arterial calcification mouse model, where a strong correlation between elastin and microcalcification content was observed.^19^

We explored potential mechanisms driving microcalcification precipitation in thoracic aneurysm disease focusing on aortopathy samples with mild calcification and mild degeneration where elastin is still intact. By selecting the early stages of disease, we have focused our immunohistochemistry experiments on processes occurring before, but not caused by, the known propagating effects of microcalcification on further calcification.^9,20^ In these early aortopathy disease samples, AP was increased and is consistent with previous studies using FBN1^C1041G/+^ murine models of the Marfan syndrome.^8^ Furthermore, we find higher OPN, which is associated with tissue damage and vascular smooth muscle cell transdifferentiation in vascular disease^21^—a result consistent with previous reports in bicuspid aortic valve aortopathy.^17^ Overall, while our results find associations between markers of smooth muscle cell transdifferentiation and AP and early medial microcalcification in aortopathy, further research is required to elucidate the specific pathways involved. For example, Krüppel-like factor 4—an upstream mediator of OPN expression in vascular smooth muscle cell osteogenic transdifferentiation^22^—has not been explored in the current work but has been reported in distinct smooth muscle cell subtypes in Marfan syndrome^23^ and represents an important research focus for exploring early microcalcification in noninflammatory thoracic aortopathy. Thus, greater characterization is needed to gain a more complete understanding of these processes.

While higher microcalcification was associated with mild and moderate histopathologic thoracic aortic aneurysm severity, we were surprised to find that severe pathological disease was associated with low levels of microcalcification, suggesting a nonlinear pathobiological course. Through biochemical and both within and between sample histological quantification, we find that this decline in microcalcification is associated with loss of elastin fibers on which the microcalcification has precipitated. While elastin fragmentation is known to have an augmenting effect on microcalcification,^8,24^ total elastin destruction or loss seen in end-stage disease appears to have the opposite effect, which will need to be accounted for when assessing histological severity by microcalcification. In line with this, we find a linear relationship between both microcalcification content and elastin content with tissue elastic modulus, suggesting biomechanical weakness in those with low microcalcification and elastin content. Supporting this, multiple previous reports have found a close relationship between intact elastin and delamination strength.^25,26^ If microcalcification could be serially visualized, it has the potential to provide crucial information about the reduction of elastin integrity over time and therefore dissection or rupture potential of thoracic aortic aneurysms.

We found that the radiotracer ^18^F-sodium fluoride, which binds specifically to hydroxyapatite (calcium phosphate crystals),^14,27^ had excellent visual colocalization with histological specimens stained with von Kossa. We present the case of a patient with significant aortopathy and focal wall weakness culminating in aortic wall outpouching (Graphic Abstract). While the overall aneurysm ^18^F-sodium fluoride uptake is high, the areas of weakness have low signal, suggesting focal elastin decay and high biomechanical failure potential. These findings provide proof of concept for ^18^F-sodium fluoride positron emission tomography imaging in detecting the vulnerable aortic wall in aortopathy. The potential applications of such imaging for those who would most benefit from surgery are 2-fold. First, thoracic aortic aneurysm microcalcification is indicative of mild or moderate disease where the elastin is still largely intact and may represent a lower risk stage in the disease that could be initially managed conservatively and monitored over time. Second, a patient with focal areas of low ^18^F-sodium fluoride uptake, as in the case presented, or one with decreasing thoracic aneurysm microcalcification over time, would signify advanced disease with poor elastin integrity that might prompt earlier intervention.

It is important to outline some limitations of the current work. All samples were obtained from tissue biobanks, and demographic information and data on comorbidities were incomplete. Specifically, control thoracic aortic tissue was obtained from heart donors and is completely deidentified and anonymized precluding any comparison of demographics to the disease groups. However, tissue from beating heart donors represents the gold standard for control thoracic aortic tissue and represents the most robust control tissue available. While our results demonstrate an important relationship between microcalcification and histopathologic severity in thoracic aortopathy, histological specimens represent a single snapshot biased toward more severe disease, and our results cannot determine the time course of disease progression. Progress in this regard will come from sequential ^18^F-sodium fluoride imaging of patients with thoracic aortopathy over time, which is currently being explored (NCT04083118).

In conclusion, we found that aortopathy is associated with increased medial microcalcification, AP, and OPN. Microcalcification precipitates on elastin fibers, peaking in mild and moderate thoracic aortic aneurysm disease but falling in severe disease in line with the decay and loss of elastin fibers. The fall in elastin is associated with reduced stiffness and represents an area of biomechanical weakness. Finally, we show that the radiotracer ^18^F-sodium fluoride colocalizes with medial microcalcification, paving the way for noninvasive assessment of thoracic aortic aneurysm disease severity, with major implications for selecting ideal candidates for elective repair.

## Article Information

### Acknowledgments

The authors acknowledge the involvement of Othman, Nawaytou, Harrington, and Kuduvalli in tissue collection and the Aortic Fellows in the consenting process at the Liverpool Heart and Chest Hospital. The authors also would like to thank Drs Ya Hua Chim and Hannah Davies for assisting with the biomechanical and biochemical analysis at Liverpool. The authors also acknowledge the assistance of Edinburgh Imaging facilities supported by the National Health Service Research Scotland through the National Health Service Lothian Health Board and Craig Marshall and the BioResource team at the Royal Infirmary of Edinburgh. For the purpose of open access, the author has applied a Creative Commons Attribution (CC-BY) licence to any author accepted manuscript version arising from this submission.

### Sources of Funding

A.J. Fletcher (FS/19/15/34155), M.G. Macaskill (RG/16/10/32375 and PG/17/83/33370), and D.E. Newby is supported by the British Heart Foundation (CH/09/002, RG/16/10/32375, and RE/18/5/34216) and is the recipient of a Wellcome Trust Senior Investigator Award (WT103782AIA). Biomechanical and biochemical assessment was supported by the British Heart Foundation to R. Akhtar and J. Madine, respectively (PG/16/107/32681 and FS/12/61/29877). A.A.S. Tavares is funded by the British Heart Foundation (FS/19/34/34354). A.A.S. Tavares is a recipient of a Wellcome Trust Technology Development Award (221295/Z/20/Z). S. Sellers is supported by a Providence Health Care Research Institute Early Career Initiative.

### Disclosures

J.A. Leipsic is a consultant for and has stock options in HeartFlow, Inc, and Circl CVI and provides computed tomography core laboratory services to Edwards Lifesciences, Medtronic, and Neovasc for which no direct compensation is received. The other authors report no conflicts.

### Supplemental Material

Supplemental Results

Figures S1–S7

Tables S1–S4

## Supplementary Material



## References

[1] Melvinsdottir IH Lund SH Agnarsson BA Sigvaldason K Gudbjartsson T Geirsson A. The incidence and mortality of acute thoracic aortic dissection: results from a whole nation study. Eur J Cardiothorac Surg. 2016;50:1111–1117. doi: 10.1093/ejcts/ezw235 27334108 10.1093/ejcts/ezw235

[2] Kalogerakos PD Zafar MA Li Y Mukherjee SK Ziganshin BA Rizzo JA Elefteriades JA. Root dilatation is more malignant than ascending aortic dilation. J Am Heart Assoc. 2021;10:e020645. doi: 10.1161/JAHA.120.020645 34238012 10.1161/JAHA.120.020645 PMC8483477

[3] Erbel R Aboyans V Boileau C Bossone E Bartolomeo RD Eggebrecht H Evangelista A Falk V Frank H Gaemperli O; ESC Committee for Practice Guidelines. 2014 ESC guidelines on the diagnosis and treatment of aortic diseases: document covering acute and chronic aortic diseases of the thoracic and abdominal aorta of the adult. The Task Force for the Diagnosis and Treatment of Aortic Diseases of the European Society of Cardiology (ESC). Eur Heart J. 2014;35:2873–2926. doi: 10.1093/eurheartj/ehu281 25173340 10.1093/eurheartj/ehu281

[4] Hiratzka LF Bakris GL Beckman JA Bersin RM Carr VF Casey DE Jr Eagle KA Hermann LK Isselbacher EM Kazerooni EA; American College of Cardiology Foundation/American Heart Association Task Force on Practice Guidelines; American Association for Thoracic Surgery; American College of Radiology; American Stroke Association; Society of Cardiovascular Anesthesiologists; Society for Cardiovascular Angiography and Interventions; Society of Interventional Radiology; Society of Thoracic Surgeons; Society for Vascular Medicine. 2010 ACCF/AHA/AATS/ACR/ASA/SCA/SCAI/SIR/STS/SVM guidelines for the diagnosis and management of patients with Thoracic Aortic Disease: a report of the American College of Cardiology Foundation/American Heart Association Task Force on Practice Guidelines, American Association for Thoracic Surgery, American College of Radiology, American Stroke Association, Society of Cardiovascular Anesthesiologists, Society for Cardiovascular Angiography and Interventions, Society of Interventional Radiology, Society of Thoracic Surgeons, and Society for Vascular Medicine. Circulation. 2010;121:e266–e369. doi: 10.1161/CIR.0b013e3181d4739e 20233780 10.1161/CIR.0b013e3181d4739e

[5] Fletcher AJ Syed MBJ Aitman TJ Newby DE Walker NL. Inherited thoracic aortic disease: new insights and translational targets. Circulation. 2020;141:1570–1587. doi: 10.1161/CIRCULATIONAHA.119.043756 32392100 10.1161/CIRCULATIONAHA.119.043756 PMC7217141

[6] Kreibich M Desai ND. Reply. Ann Thorac Surg. 2020;109:614–615. doi: 10.1016/j.athoracsur.2019.07.059 10.1016/j.athoracsur.2019.07.059 31521593

[7] Haunschild J Schellinger IN von Salisch S Bakhtiary F Misfeld M Mohr FW Raaz U Etz CD. Granular media calcinosis in the aortic walls of patients with bicuspid and tricuspid aortic valves. Ann Thorac Surg. 2017;103:1178–1185. doi: 10.1016/j.athoracsur.2016.07.018 27666780 10.1016/j.athoracsur.2016.07.018

[8] Wanga S Hibender S Ridwan Y van Roomen C Vos M van der Made I van Vliet N Franken R van Riel LA Groenink M. Aortic microcalcification is associated with elastin fragmentation in Marfan syndrome. J Pathol. 2017;243:294–306. doi: 10.1002/path.4949 28727149 10.1002/path.4949

[9] Lei Y Sinha A Nosoudi N Grover A Vyavahare N. Hydroxyapatite and calcified elastin induce osteoblast-like differentiation in rat aortic smooth muscle cells. Exp Cell Res. 2014;323:198–208. doi: 10.1016/j.yexcr.2014.01.011 24447384 10.1016/j.yexcr.2014.01.011 PMC3969787

[10] O’Leary SA Mulvihill JJ Barrett HE Kavanagh EG Walsh MT McGloughlin TM Doyle BJ. Determining the influence of calcification on the failure properties of abdominal aortic aneurysm (AAA) tissue. J Mech Behav Biomed Mater. 2015;42:154–167. doi: 10.1016/j.jmbbm.2014.11.005 25482218 10.1016/j.jmbbm.2014.11.005

[11] Forsythe RO Dweck MR McBride OMB Vesey AT Semple SI Shah ASV Adamson PD Wallace WA Kaczynski J Ho W. 18F-sodium fluoride uptake in abdominal aortic aneurysms: the SoFIA3 study. J Am Coll Cardiol. 2018;71:513–523. doi: 10.1016/j.jacc.2017.11.053 29406857 10.1016/j.jacc.2017.11.053 PMC5800891

[12] Hiratzka LF Bakris GL Beckman JA Bersin RM Carr VF Casey DE Jr Eagle KA Hermann LK Isselbacher EM Kazerooni EA; American College of Cardiology Foundation/American Heart Association Task Force on Practice Guidelines; American Association for Thoracic Surgery; American College of Radiology; American Stroke Association; Society of Cardiovascular Anesthesiologists; Society for Cardiovascular Angiography and Interventions; Society of Interventional Radiology; Society of Thoracic Surgeons; Society for Vascular Medicine. 2010 ACCF/AHA/AATS/ACR/ASA/SCA/SCAI/SIR/STS/SVM guidelines for the diagnosis and management of patients with thoracic aortic disease. A Report of the American College of Cardiology Foundation/American Heart Association Task Force on Practice Guidelines, American Association for Thoracic Surgery, American College of Radiology,American Stroke Association, Society of Cardiovascular Anesthesiologists, Society for Cardiovascular Angiography and Interventions, Society of Interventional Radiology, Society of Thoracic Surgeons,and Society for Vascular Medicine. J Am Coll Cardiol. 2010;55:e27–e129. doi: 10.1016/j.jacc.2010.02.015 20359588 10.1016/j.jacc.2010.02.015

[13] Halushka MK Angelini A Bartoloni G Basso C Batoroeva L Bruneval P Buja LM Butany J d’Amati G Fallon JT. Consensus statement on surgical pathology of the aorta from the Society for Cardiovascular Pathology and the Association For European Cardiovascular Pathology: II. Noninflammatory degenerative diseases - nomenclature and diagnostic criteria. Cardiovasc Pathol. 2016;25:247–257. doi: 10.1016/j.carpath.2016.03.002 27031798 10.1016/j.carpath.2016.03.002

[14] Moss AJ Sim AM Adamson PD Seidman MA Andrews JPM Doris MK Shah ASV BouHaidar R Alcaide-Corral CJ Williams MC. Ex vivo 18F-fluoride uptake and hydroxyapatite deposition in human coronary atherosclerosis. Sci Rep. 2020;10:20172. doi: 10.1038/s41598-020-77391-6 33214599 10.1038/s41598-020-77391-6 PMC7677392

[15] Akhtar R Drapper E Adams D Hay J. Oscillatory nanoindentation of highly compliant hydrogels: a critical comparative analysis with rheometry. J Mater Res. 2018;33:873–883. doi: 10.1557/jmr.2018.62

[16] Chim YH Davies HA Mason D Nawaytou O Field M Madine J Akhtar R. Bicuspid valve aortopathy is associated with distinct patterns of matrix degradation. J Thorac Cardiovasc Surg. 2020;160:e239–e257. doi: 10.1016/j.jtcvs.2019.08.094 31679706 10.1016/j.jtcvs.2019.08.094 PMC7674632

[17] Stern C Scharinger B Tuerkcan A Nebert C Mimler T Baranyi U Doppler C Aschacher T Andreas M Stelzmueller ME. Strong signs for a weak wall in tricuspid aortic valve associated aneurysms and a role for osteopontin in bicuspid aortic valve associated aneurysms. Int J Mol Sci. 2019;20:E4782. doi: 10.3390/ijms20194782 31561491 10.3390/ijms20194782 PMC6802355

[18] Stone JR Bruneval P Angelini A Bartoloni G Basso C Batoroeva L Buja LM Butany J d’Amati G Fallon JT. Consensus statement on surgical pathology of the aorta from the Society for Cardiovascular Pathology and the Association for European Cardiovascular Pathology: I. Inflammatory diseases. Cardiovasc Pathol. 2015;24:267–278. doi: 10.1016/j.carpath.2015.05.001 26051917 10.1016/j.carpath.2015.05.001

[19] Khavandgar Z Roman H Li J Lee S Vali H Brinckmann J Davis EC Murshed M. Elastin haploinsufficiency impedes the progression of arterial calcification in MGP-deficient mice. J Bone Miner Res. 2014;29:327–337. doi: 10.1002/jbmr.2039 23857752 10.1002/jbmr.2039

[20] Ewence AE Bootman M Roderick HL Skepper JN McCarthy G Epple M Neumann M Shanahan CM Proudfoot D. Calcium phosphate crystals induce cell death in human vascular smooth muscle cells: a potential mechanism in atherosclerotic plaque destabilization. Circ Res. 2008;103:e28–e34. doi: 10.1161/CIRCRESAHA.108.181305 18669918 10.1161/CIRCRESAHA.108.181305

[21] Lok ZSY Lyle AN. Osteopontin in vascular disease. Arterioscler Thromb Vasc Biol. 2019;39:613–622. doi: 10.1161/ATVBAHA.118.311577 30727754 10.1161/ATVBAHA.118.311577 PMC6436981

[22] Yoshida T Yamashita M Hayashi M. Kruppel-like factor 4 contributes to high phosphate-induced phenotypic switching of vascular smooth muscle cells into osteogenic cells. J Biol Chem. 2012;287:25706–25714. doi: 10.1074/jbc.M112.361360 22679022 10.1074/jbc.M112.361360 PMC3406659

[23] Pedroza AJ Tashima Y Shad R Cheng P Wirka R Churovich S Nakamura K Yokoyama N Cui JZ Iosef C. Single-cell transcriptomic profiling of vascular smooth muscle cell phenotype modulation in marfan syndrome aortic aneurysm. Arterioscler Thromb Vasc Biol. 2020;40:2195–2211. doi: 10.1161/ATVBAHA.120.314670 32698686 10.1161/ATVBAHA.120.314670 PMC7484233

[24] Hosaka N Mizobuchi M Ogata H Kumata C Kondo F Koiwa F Kinugasa E Akizawa T. Elastin degradation accelerates phosphate-induced mineralization of vascular smooth muscle cells. Calcif Tissue Int. 2009;85:523–529. doi: 10.1007/s00223-009-9297-8 19806384 10.1007/s00223-009-9297-8

[25] Chung JC Wong E Tang M Eliathamby D Forbes TL Butany J Simmons CA Ouzounian M. Biomechanics of aortic dissection: a comparison of aortas associated with bicuspid and tricuspid aortic valves. J Am Heart Assoc. 2020;9:e016715. doi: 10.1161/JAHA.120.016715 32750292 10.1161/JAHA.120.016715 PMC7792273

[26] Tam AS Sapp MC Roach MR. The effect of tear depth on the propagation of aortic dissections in isolated porcine thoracic aorta. J Biomech. 1998;31:673–676. doi: 10.1016/s0021-9290(98)00058-x 9796691 10.1016/s0021-9290(98)00058-x

[27] Irkle A Vesey AT Lewis DY Skepper JN Bird JL Dweck MR Joshi FR Gallagher FA Warburton EA Bennett MR. Identifying active vascular microcalcification by (18)F-sodium fluoride positron emission tomography. Nat Commun. 2015;6:7495. doi: 10.1038/ncomms8495 26151378 10.1038/ncomms8495 PMC4506997

