# Supplementary material for: Microcalcification and Thoracic Aortopathy: A Window Into Disease Severity
Source: Arterioscler Thromb Vasc Biol. 2022 Jun 30;42(8):1048–59. doi: 10.1161/ATVBAHA.122.317111 (PMC9311465; doi:10.1161/ATVBAHA.122.317111)
Supplement: Supplementary file 1 [file atv-42-1048-s001.pdf]

Supplementary Material

Microcalcification and thoracic aortopathy: a window into disease severity

Alexander J Fletcher,[a] Jennifer Nash,[a] Maaz Syed,[a] Mark G Macaskill,[b] Adriana A S Tavares,[b] Niki Walker,[a,c] Hannah Salcudean,[d] Jonathon A. Leipsic,[d] Kelvin Lim,[e] Jillian Madine,[f,g] William Wallace,[h] Mark Field,[f,i] David E Newby, [a] Rihab Bouchareb,[j] Michael Seidman, [k] Riaz Akhtar,[f] Stephanie Sellers[d]

[a] British Heart Foundation Centre for Cardiovascular Science, University of Edinburgh, Edinburgh, United Kingdom
[b] Edinburgh Imaging Facility, Queens Medical Research Institute, University of Edinburgh, Edinburgh, United Kingdom
[c] Scottish Adult Congenital Cardiology Service, Golden Jubilee National Hospital, Clydebank, Glasgow, United Kingdom
[d] Department of Radiology, Division of Cardiology, and Cardiovascular Translational Lab at the Centre for Heart Lung Innovation, St Paul's Hospital and University of British Columbia, Vancouver, Canada
[e] Department of Cardiothoracic surgery, Royal Infirmary of Edinburgh, United Kingdom
[f] Institute of Systems, Molecular and Integrative Biology, Faculty of Health and Life Sciences, University of Liverpool, Liverpool, United Kingdom
[g] Liverpool Centre for Cardiovascular Sciences, University of Liverpool, Liverpool, United Kingdom
[h] Division of Pathology, University of Edinburgh, Edinburgh, UK
[i] Dept of Cardiothoracic Surgery, Liverpool Heart and Chest Hospital and 'f' (LCCS)
[j] Cardiovascular Research Institute, Department of medicine, The Icahn School of Medicine at Mount Sinai, New York, United States of America
[k] Department of Laboratory Medicine & Pathobiology, Toronto General Hospital, Canada
[J] Department of Mechanical, Materials and Aerospace Engineering, School of Engineering, University of Liverpool, Liverpool, United Kingdom

Major Resources Table

In order to allow validation and replication of experiments, all essential research materials listed in the Methods should be included in the Major Resources Table below. Authors are encouraged to use public repositories for protocols, data, code, and other materials and provide persistent identifiers and/or links to repositories when available. Authors may add or delete rows as needed.

Antibodies

Target antigen	Vendor or Source	Catalog #	Working concentration	Persistent ID / URL
Osteopontin	Sigma-Aldrich	O7264	5µg/ml (1:200 of 1mg/ml)	https://www.sigmaaldrich.com/GB/en/product/sigma/o7264
Cleaved Caspase III	Cell Signaling	9664	0.4µg/ml (1:100 of 40 0.4µg/ml)	https://www.cellsignal.com/products/primary-antibodies/cleaved-caspase-3-asp175-5a1e-rabbit-mab/9664
Alkaline phosphatase	Abcam	ab95462	1:300*	https://www.abcam.com/Alkaline-Phosphatase-antibody-ab95462.html
ERK	Cell Signaling	9102	0.4µg/ml (1:50 of 19µg/ml)	https://www.cellsignal.com/products/primary-antibodies/p44-42-mapk-erk1-2-antibody/9102
BMP2	Bio-Rad	AHP 960	1µg/ml (1:100 of 100µg/ml)	https://www.bio-rad-antibodies.com/polyclonal/human-bmp-2-antibody-ahp960.html?f=purified
Runx2	LSBio	LS-C385968	10µg/ml	https://www.lsbio.com/antibodies/cbfa1-antibody-runx2-antibody-internal-elisa-ihc-wb-western-ls-c385968/398069
WNT3a	Abcam	Ab28472	5µg/ml (1:200 of 1mg/ml)	https://www.abcam.com/Wnt3a-antibody-ab28472.html

*whole antiserum, manufacturer does not provide concentration and recommends working dilutions

Supplementary Results

Aortic wall microcalcification is associated with aortic valve disease

Across 57 patients with thoracic aortopathy, univariable linear regression analysis demonstrated that higher microcalcification content (percentage area) was associated with moderate (ß=17.38, p=0.011) and severe (ß=5.91, p=0.0496) aortic valve stenosis, whereas mild (ß= -8.37, p=0.028), moderate (ß= -9.14, p=0.0486) and severe (ß= -7.27, p=0.049) aortic regurgitation were associated with lower microcalcification compared to those without valve disease. There were no associations between age, sex, presence of bicuspid aortic valve, smoking, hypercholesterolaemia, family history, hypertension or aortic diameters and microcalcification content (all p>0.05, Supplementary Table 4).

Figure S1
Representative staining of alkaline phosphatase (ALP), osteopontin (OPN) and caspase III (Cas3) for each of the four semi-quantitive scoring categories.

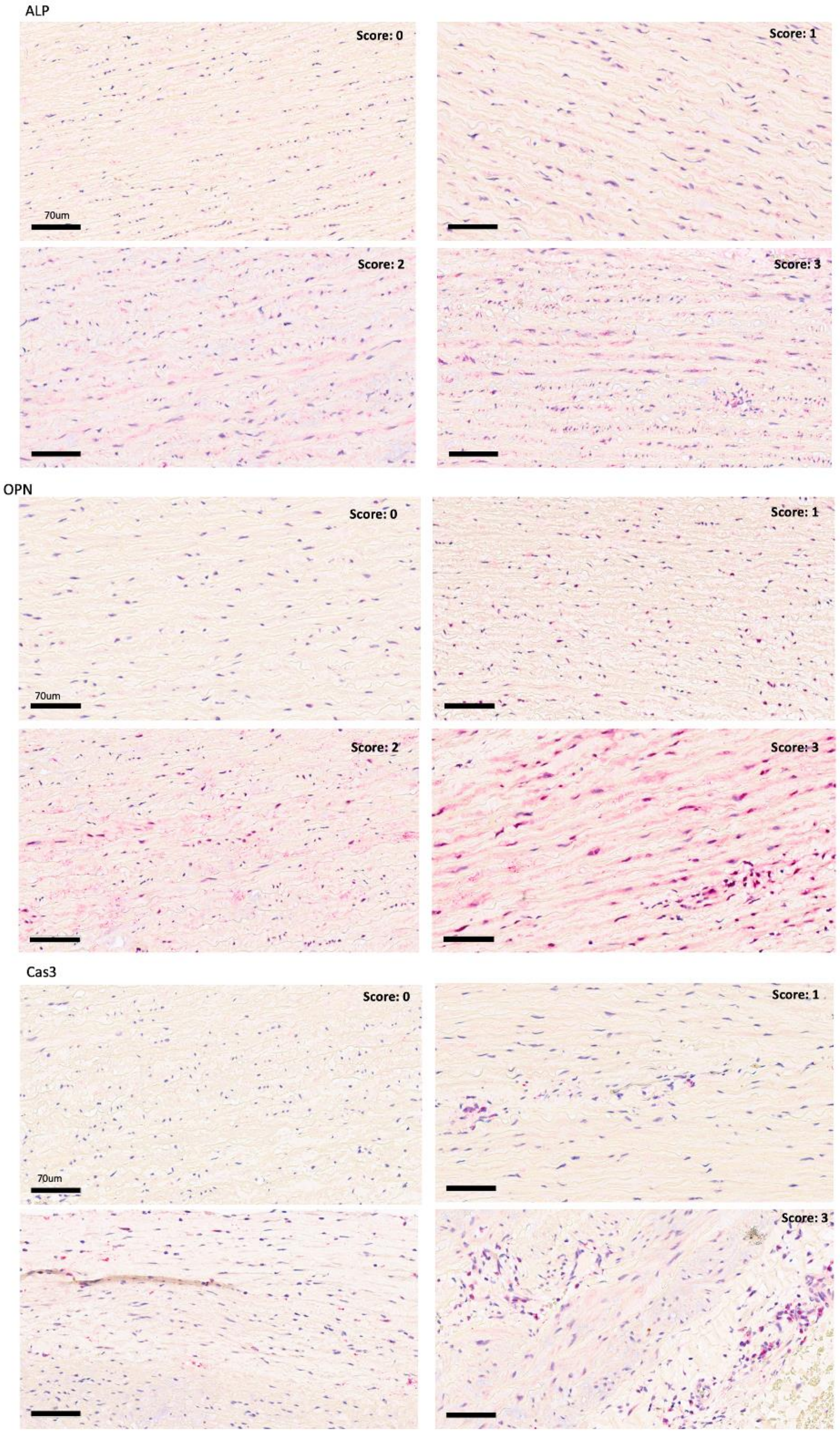

Figure S2

Examples of microcalcification quantification on images of samples stained with von Kossa.

Thresholding at 50% of total sample opacity then windowing within 10% to achieve visually optimal coverage of von Kossa staining. Microcalcification severity categories are divided into minimal, mild, moderate and severe.

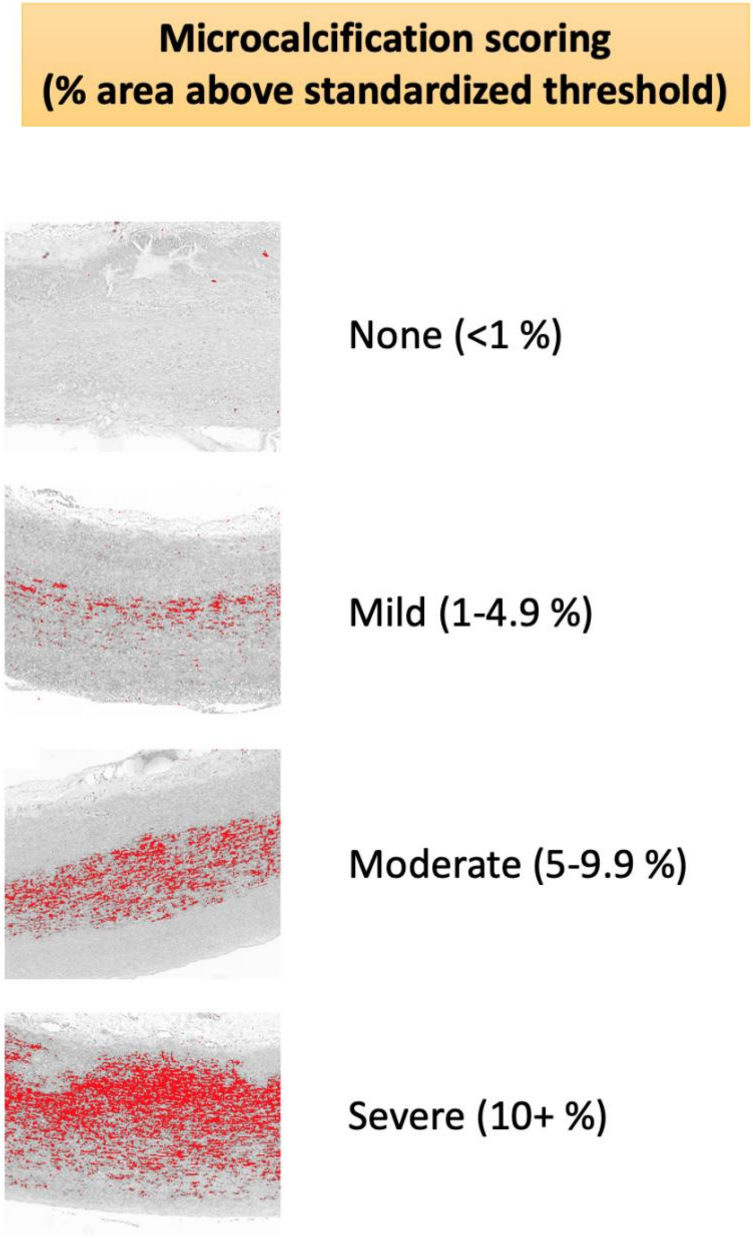

Figure S3
Intra-rater repeatability of the percentage area microcalcification quantification.
Excellent repeatability, with an interclass correlation co-efficient of 0.97 and co-efficient of repeatability of 4.

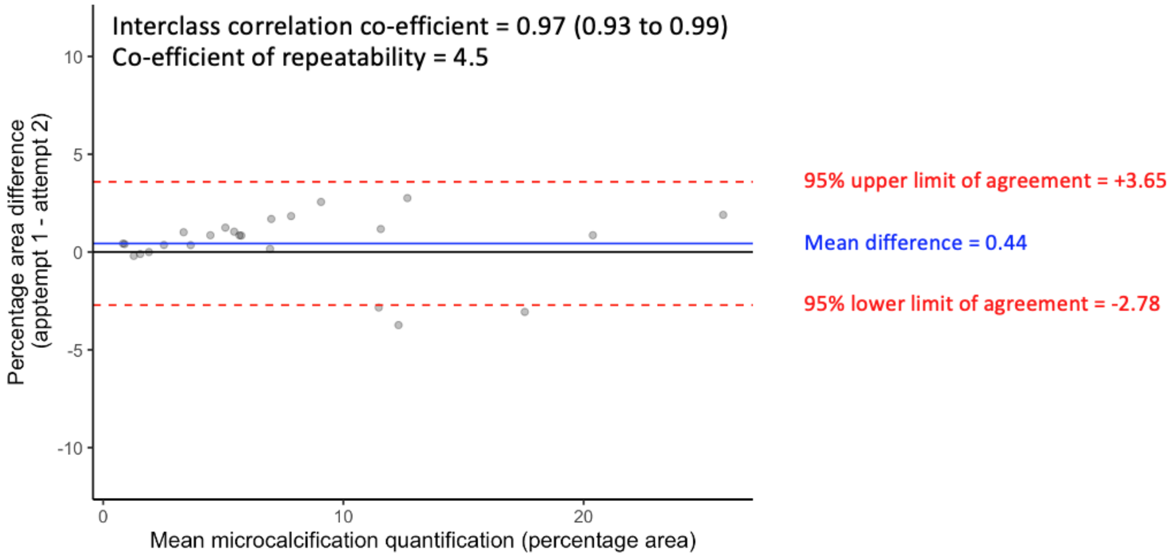

Figure S4

Control aortic tissue demonstrating healthy appearing elastin with minimal calcification (white, A) confirmed on X-ray dispersion element analysis with low levels of calcium and phosphate (B). More calcification is seen in a patient with mild histopathological aortopathy and intact elastin (C), confirmed on X-ray dispersion element analysis with high concentrations of calcium and phosphate in a 2:1 ratio signifying hydroxyapatite (D). Weight % = 100 * weight of one component (element) / weight of entire sample. Atomic % = 100 * number of atoms of one component / total number of all atoms in sample.

Control without aortopathy

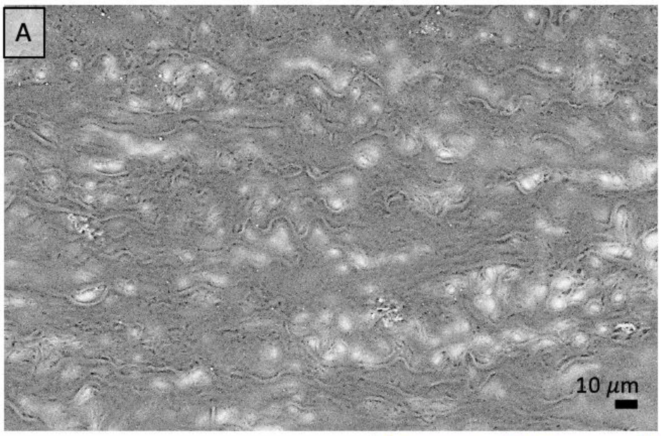

B

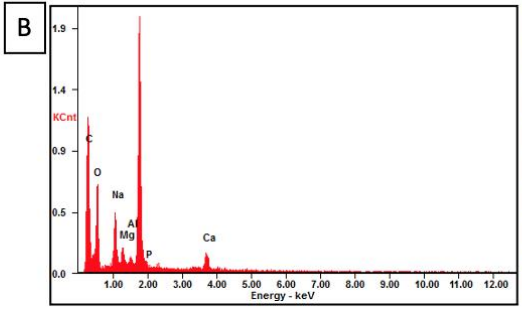

Element	Wt%	At%
CK	50.50	63.54
OK	24.41	23.06
NaK	04.23	02.78
MgK	01.23	00.76
AlK	00.35	00.20
SiK	14.09	07.58
PK	01.03	00.50
CaK	04.17	01.57
Matrix	Correction	ZAF

Mild Histological Aortopathy Severity

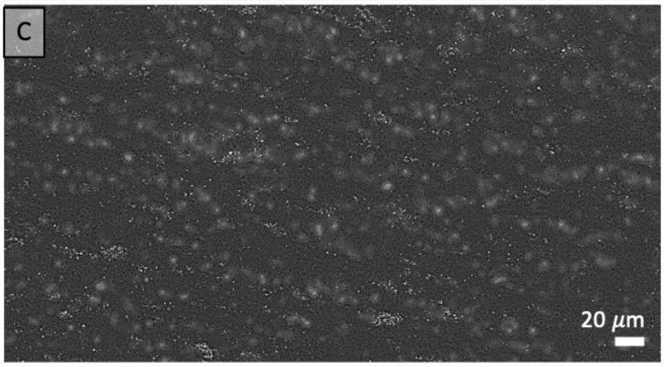

D

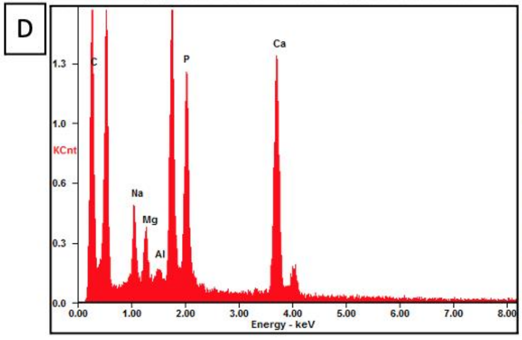

Element	Wt%	At%
CK	41.44	56.70
OK	25.97	26.68
NaK	02.24	01.60
MgK	01.27	00.86
AlK	00.22	00.14
SiK	07.46	04.36
PK	07.33	03.89
CaK	14.07	05.77
Matrix	Correction	ZAF

Figure S5
Aortic tissue from a patient with moderate histopathological aortopathy with substantial microcalcification precipitating along grossly intact elastin fibres (orange arrows, A). A magnified area of intense microcalcification deposition demonstrates varying sizes of hydroxyapatite crystal as it starts to coalescence (B), confirmed as hydroxyapatite on energy X-ray dispersion element analysis. In severe histopathological severity (D-F), a similar pattern of microcalcification precipitation along intact elastin fibres is seen (orange arrows), however areas of total elastin loss are devoid of microcalcification (yellow arrows), with X-ray dispersion confirming lower overall hydroxyapatite content (F).

Moderate Histological Aortopathy Severity

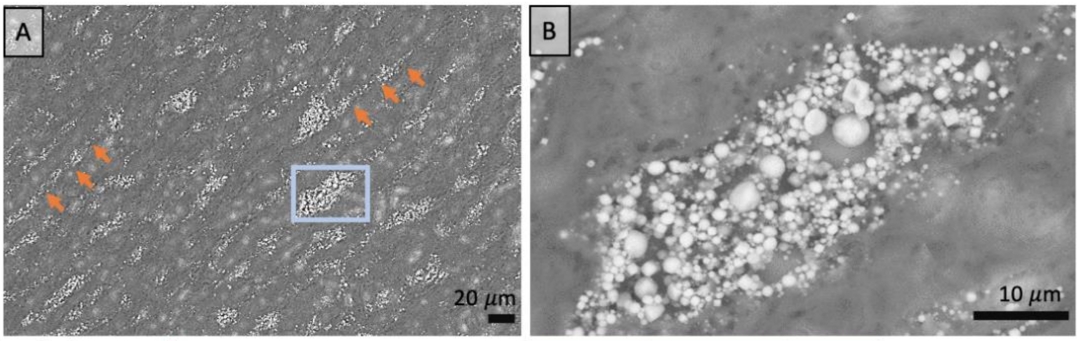

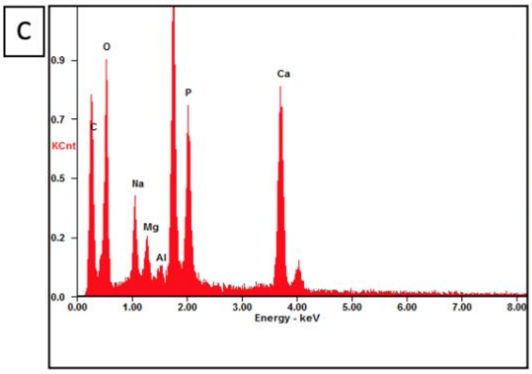

Element	Wt%	At%
CK	50.45	65.22
OK	21.29	20.66
NaK	02.65	01.79
MgK	01.04	00.66
AlK	00.30	00.17
SiK	09.50	05.25
PK	04.56	02.28
CaK	10.21	03.96
Matrix	Correction	ZAF

Severe Histological Aortopathy Severity

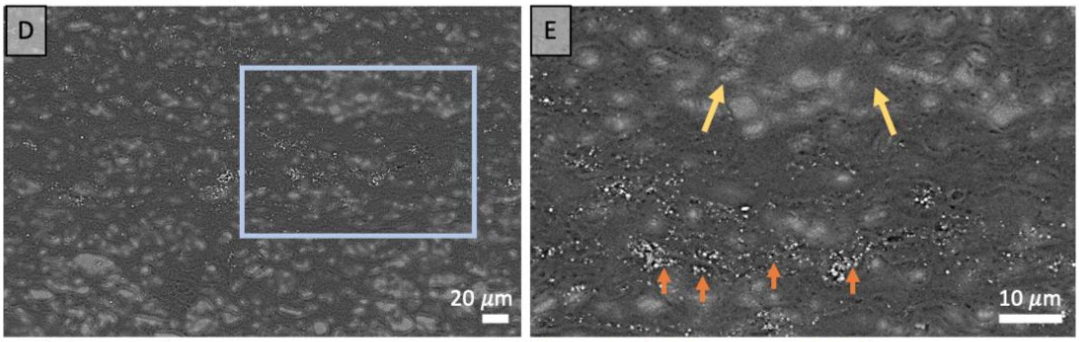

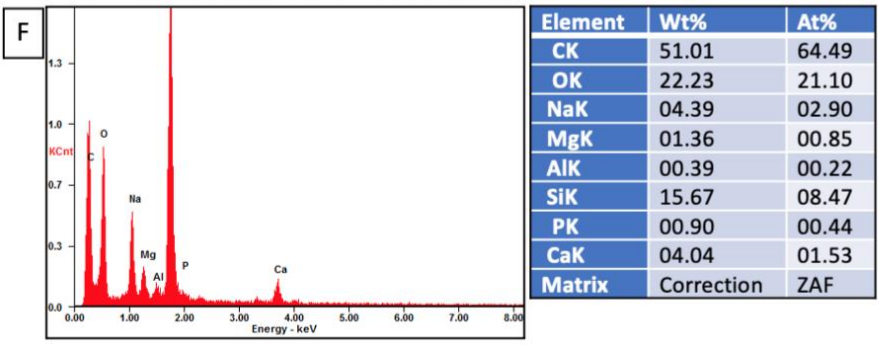

Element	Wt%	At%
CK	51.01	64.49
OK	22.23	21.10
NaK	04.39	02.90
MgK	01.36	00.85
AlK	00.39	00.22
SiK	15.67	08.47
PK	00.90	00.44
CaK	04.04	01.53
Matrix	Correction	ZAF

Figure S6
Within sample analysis in samples with discrete areas of both high and low microcalcification (A). Elastin content is higher in discrete areas of high microcalcification. (B-G) Representative example of an aortopathy sample with clear areas of high (C&F) and low (D&G) microcalcification.

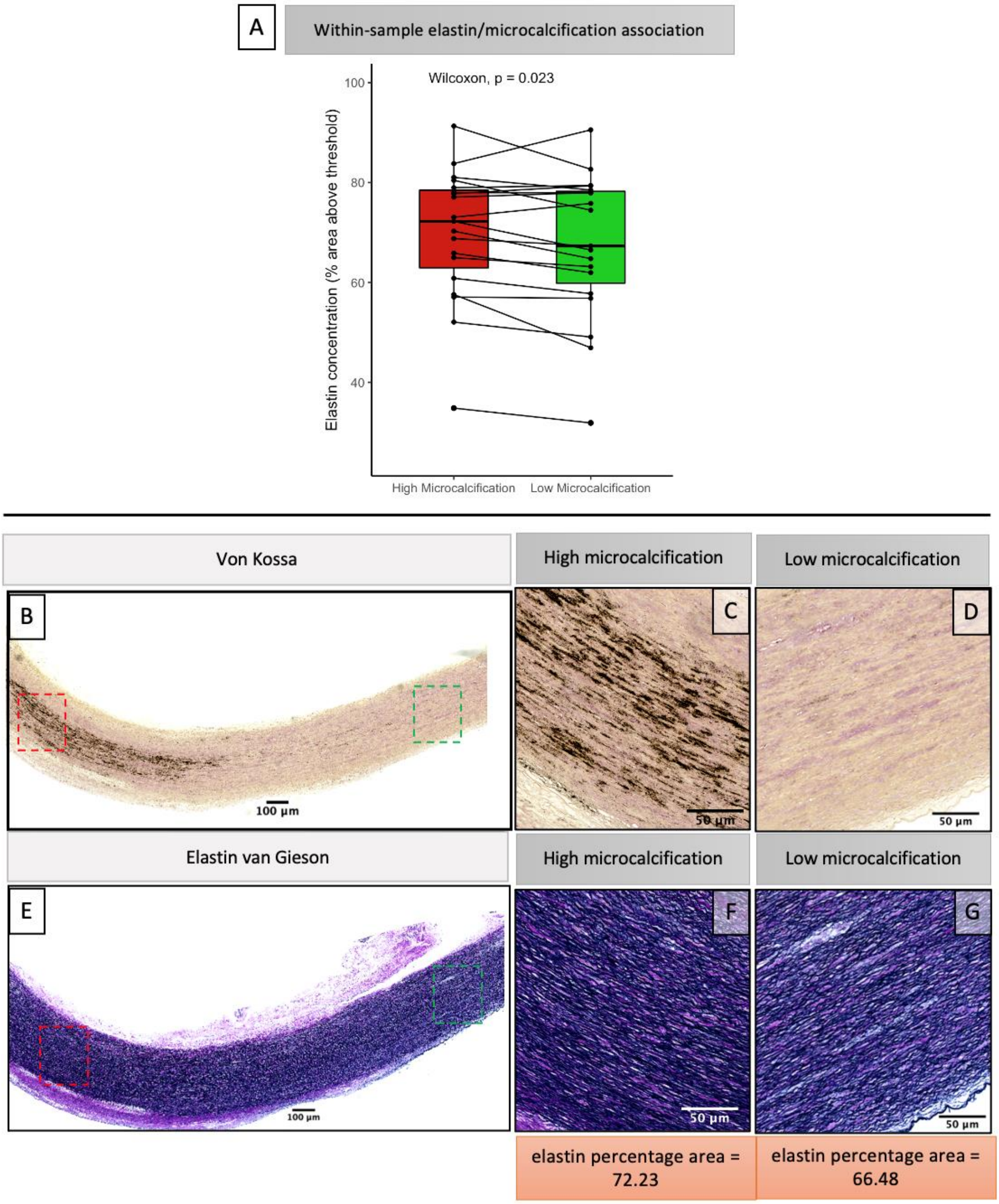

Figure S7

(A-D) representative staining of osteopontin (A&B) and microcalcification (von Kossa staining, C&D) demonstrating co-localization between osteopontin positive staining and dense microcalcification precipitation in tricuspid aortic valve thoracic aortopathy sample.

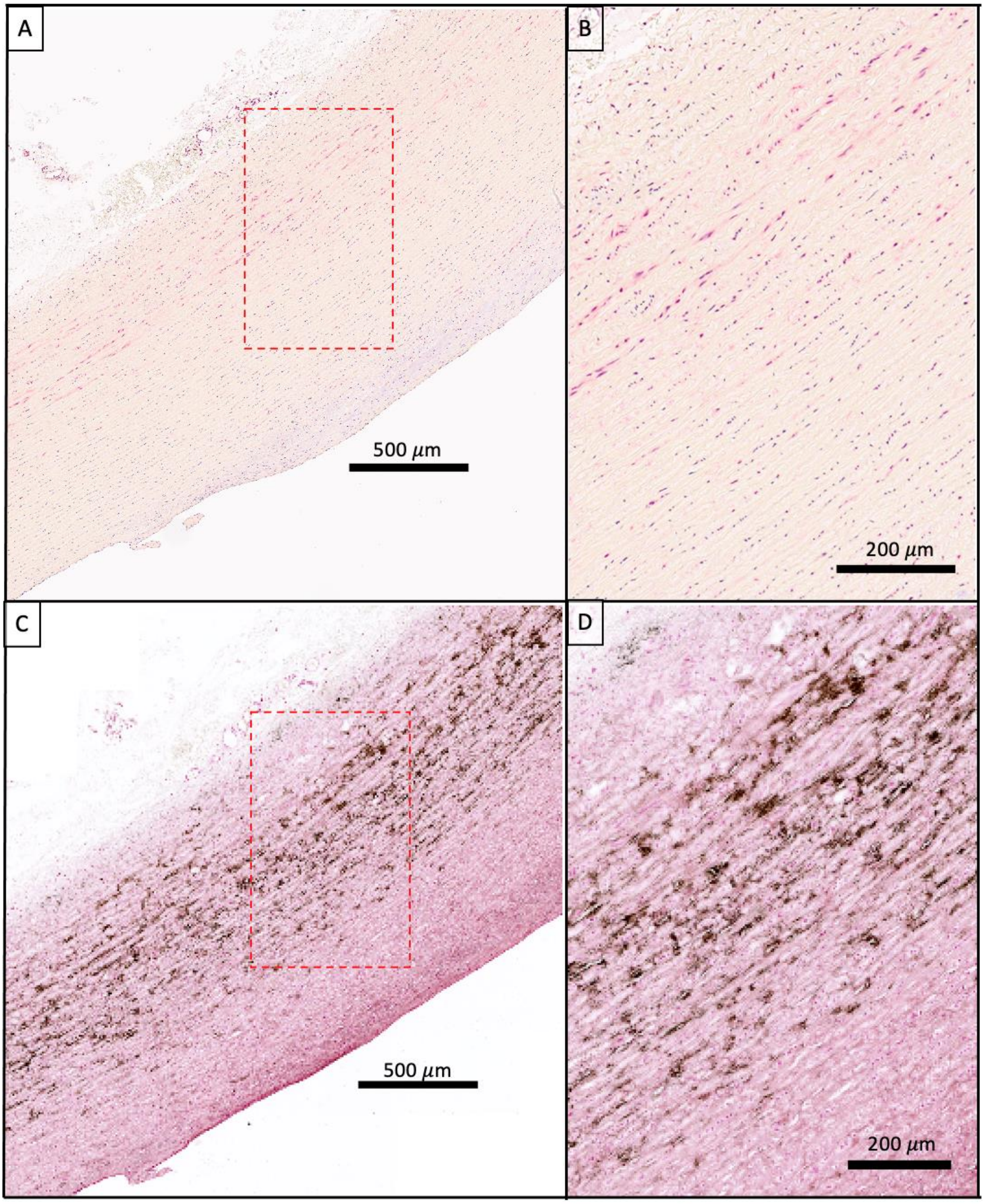

Table S1. Pathology score stratified by patient cohort

	Bicuspid aortic valve (n=41)	Tricuspid aortic valve (n=23)	Thoracic aortic dissection (n=18)	Between group (X^2) p-value
**Overall score**	5.00 [4.00 to 6.00]	6.00 [4.00 to 10.50]	8.50 [6.25 to 9.00]	0.001
**Atheroma presence**	16 (39.0)	9 (39.1)	7 (50.0)	0.645
**Intra-lamellar mucoid extracellular matrix accumulation**				<0.001
Absent	0	0	0	
Mild	18 (43.9)	8 (34.8)	0 (0.0)	
Moderate	18 (43.9)	8 (34.8)	6 (33.3)	
Severe	5 (12.2)	7 (30.4)	12 (66.7)	
**Trans-lamellar mucoid extracellular matrix accumulation**				0.064
Absent	23 (56.1)	8 (34.8)	6 (33.3)	
Moderate	15 (36.6)	9 (39.1)	8 (44.4)	
Severe	3 (7.3)	6 (26.1)	4 (22.2)	
**Elastin fragmentation or loss**				<0.001
Absent	0 (0.0)	0 (0.0)	1 (5.6)	
Mild	31 (75.6)	10 (45.5)	3 (16.7)	
Moderate	7 (17.1)	4 (18.2)	8 (44.4)	
Severe	3 (7.3)	8 (36.4)	6 (33.3)	
**Smooth muscle cell loss**				0.189
Absent	1 (2.4)	0 (0.0)	0 (0.0)	
Mild	38 (92.7)	21 (95.5)	16 (88.9)	
Moderate	2 (4.9)	1 (4.5)	0 (0.0)	
Severe	0 (0.0)	0 (0.0)	2 (11.1)	
**Medial collapse**				0.389
Absent	33 (80.5)	12 (54.5)	12 (66.7)	
Mild	7 (17.1)	7 (31.8)	6 (33.3)	
Moderate	1 (2.4)	2 (9.1)	0 (0.0)	
Severe	0 (0.0)	1 (4.5)	0 (0.0)	

Data presented as median [inter-quartile range] or number (percentage)

Table S2. Categorical repeatability of microcalcification quantification

	Microcalcification category Assessment 1 (n=24)	Microcalcification category Assessment 2 (n=24)
**Minimal - 0 to 0.99%**	0 (0.0)	2 (8.3)
**Mild - 1.00 to 4.99%**	9 (37.5)	9 (37.5)
**Moderate - 5.00 to 9.99%**	7 (29.2)	6 (25.0)
**Severe - ≥10%**	8 (33.3)	7 (29.2)

Table S3. Semi-quantitative immunohistochemistry scores for each biomarker assessed

	Aortopathy (n=42)	Control (n=7)	p-value (χ^2)
**Caspase III Intima**			**0.569**
Minimal	32 (76.2)	5 (71.4)	
Mild	5 (11.9)	2 (28.6)	
Moderate	2 (4.8)	0 (0.0)	
Severe	3 (7.1)	0 (0.0)	
**Caspase III Media**			**0.266**
Minimal	26 (61.9)	7 (100.0)	
Mild	14 (33.3)	0 (0.0)	
Moderate	1 (2.4)	0 (0.0)	
Severe	1 (2.4)	0 (0.0)	
**Osteopontin**			**0.001**
Minimal	9 (21.4)	7 (100.0)	
Mild	13 (31.0)	0 (0.0)	
Moderate	11 (26.2)	0 (0.0)	
Severe	9 (21.4)	0 (0.0)	
**p-ERK**			**0.138**
Minimal	11 (26.2)	4 (66.7)	
Mild	12 (28.6)	2 (33.3)	
Moderate	14 (33.3)	0 (0.0)	
Severe	5 (11.9)	0 (0.0)	

Table S4. Patient-level associations with thoracic aortic microcalcification content

	ß-estimate	Standard error	Intercept	p-value
**Age**	0.09	0.073	0.089	0.22
**Sex**	0.32	1.87	5.45	0.86
**Current smoker**	-0.89	2.54	6.17	0.73
**Family history aortopathy**	-2.56	2.91	5.98	0.38
**Hypertension**	-0.88	1.88	6.22	0.64
**Hyperlipidaemia**	3.85	1.93	3.88	0.053
**Bicuspid aortic valve**	2.835	1.96	4.39	0.15
**Aortic Stenosis**				
Mild	2.06	4.53	2.02	0.66
Moderate	17.38	6.07	2.02	0.011
Severe	5.91	2.78	2.02	0.0496
**Aortic Regurgitation**				
Mild	-8.37	3.79	9.84	0.042
Moderate	-9.14	3.79	9.84	0.028
Severe	-7.27	3.41	9.84	0.0486
**Aortic root diameter**	-0.03	0.22	6.42	0.89
**Ascending aortic diameter**	0.009	0.14	5.74	0.95
**Maximal aortic diameter**	-0.16	0.098	14.48	0.098
